# 101. Effect of Human Papillomavirus Vaccine to Interrupt Recurrence of Vulvar and Anal Neoplasia (VIVA): A Randomized, Placebo-Controlled Trial

**DOI:** 10.1093/ofid/ofac492.179

**Published:** 2022-12-15

**Authors:** Helen C Stankiewicz Karita, Amalia Magaret, Amalia Magaret, Jeffrey Schouten, Constance Mao, Warner Huh, Verena Grieco, Matthew Seymour, Dana Varon, David Doody, Long Fu xi, Denise Galloway, Anna Wald, Margaret Madeleine

**Affiliations:** University of Washington, Seattle, Washington; University of Washington, Seattle, Washington; University of Washington, Seattle, Washington; University of Washington, Seattle, Washington; University of Washington, Seattle, Washington; University of Alabama at Birmingham, Birmingham, Washington; University of Washington, Seattle, Washington; University of Washington, Seattle, Washington; University of Washington, Seattle, Washington; University of Washington, Seattle, Washington; University of Washington, Seattle, Washington; University of Washington, Seattle, Washington; University of Washington, Seattle, Washington; University of Washington, Seattle, Washington

## Abstract

**Background:**

Anal and vulvar high-grade intraepithelial lesions (HSIL) often recur after primary treatment, with 30%-50% recurrence in the 5-years following treatment. Treatment for recurrent lesions can be uncomfortable, debilitating, and costly. The VIVA trial evaluated the effects of the nonavalent human papillomavirus (HPV) vaccine (9vHPV) on recurrent anal or vulvar HSIL.

**Methods:**

We conducted a randomized, double-blinded, placebo-controlled trial of the 9vHPV in vaccine-naïve persons aged 27-69 years who were previously treated for anal or vulvar HSIL and HSIL-free at enrollment. Participants had high-resolution anoscopy or vulvoscopy at screening, month 18 and 36 visits. Eligible participants were randomly assigned (1:1) to receive 9vHPV or placebo on day 1, month 2 and 6. We hypothesized that 9vHPV leads to a 50% reduction of HSIL recurrence. The primary endpoint was anal or vulvar HSIL recurrence, which was assessed in the intent-to-treat (ITT) population.

**Results:**

Between July 2017 and December 2021, 187participants (99 cis-men, 86 cis-women, 2 transgender persons) with a history of anal (104, 56%) or vulvar (83, 44%) HSIL enrolled in the trial. 181 (97%) participants were included in the ITT analysis. Median age was 55 years (IQR 48-63); 71 out of 181 participants (39%) had well-controlled HIV infection. The DSMB recommended stopping the study early because it met specified futility boundaries at interim analysis. Predictive power to show a significant difference in the primary endpoint was 6.4% should the study continue to accrue. With 46% of planned information accrued, the vaccine was not significantly more efficacious than the placebo in preventing recurrent HSIL, with 15 cases in the 9vHPV arm versus 18 cases in placebo (incidence 9.1 versus 10.0/100 person-years; p= 0.83 by log-rank test). We found no differences in HSIL recurrences among vaccine versus placebo recipients by anatomical site or HIV status. The 9vHPV was safe and well-tolerated.

Kaplan-Meier Curve for overall high-grade squamous intra-epithelial neoplasia (HSIL) recurrences for nonavalent-HPV vaccine vs placebo recipients

**Figure:**
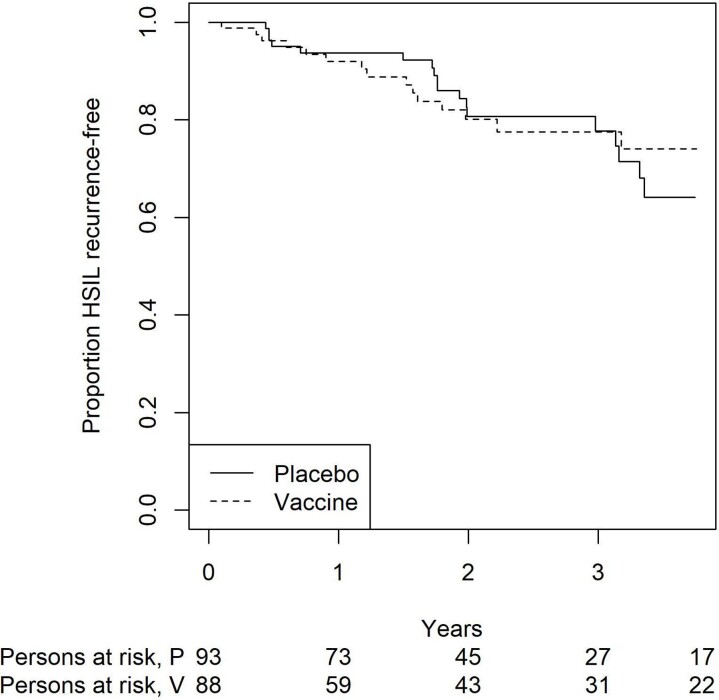

**Conclusion:**

We found no benefit of the 9vHPV vaccine for prevention of recurrent anal or vulvar HSIL. Our study underlines the importance of HPV prevention with prophylactic HPV vaccines and the need for novel therapeutic vaccines and antivirals to manage prevalent HSIL that have a high potential to recur.

**Disclosures:**

**Denise Galloway, PhD**, Merck: Grant/Research Support **Anna Wald, MD, MPH**, Aicuris: Advisor/Consultant|Auritec: Advisor/Consultant|Crozet: Advisor/Consultant|DXNow: Advisor/Consultant|GSK: Grant/Research Support|Merck: Advisor/Consultant|sanofi: Grant/Research Support|VIR: Advisor/Consultant|X-Vax: Advisor/Consultant.

